# A Mixed-Methods Approach for Evaluating Implementation Processes and Program Costs for a Hypertension Management Program Implemented in a Federally Qualified Health Center

**DOI:** 10.1007/s11121-023-01529-x

**Published:** 2023-06-30

**Authors:** Aisha Tucker-Brown, Michelle Spafford, John Wittenborn, David Rein, Ashley Marshall, Kincaid Lowe Beasley, Marla Vaughan, Natalie Nelson, Michelle Dougherty, Roy Ahn

**Affiliations:** 1 Division for Heart Disease and Stroke Prevention, Centers for Disease Control and Prevention, 4770 Buford Highway NE, GA 30341 Atlanta, USA; 2 Health Care Evaluation Department, NORC at the University of Chicago, 4350 East-West Highway, 8th Floor, MD 20814 Bethesda, USA; 3 Public Health Department, NORC at the University of Chicago, 55 East Monroe, 31st Floor, IL 60603 Chicago, USA; 4 Public Health Department, NORC at the University of Chicago, 1447 Peachtree Street NE, Suite 700, Atlanta, GA 30309 USA; 5 Division of Laboratory Systems (DLS), Centers for Disease Control and Prevention, 2400 Century Center, Atlanta, GA 30345 United States; 6 Family Health Centers, Inc, 3310 Magnolia St, Orangeburg, SC 29115 USA; 7 Public Health Department, NORC at the University of Chicago, 4350 East-West Highway, 8th Floor, MD 20814 Bethesda, USA

**Keywords:** Evaluation, Hypertension, Team-based care, Federally qualified health center, Pharmacy

## Abstract

Team-based care approaches are effective at improving hypertension control and have been used in clinical practice to improve hypertension outcomes. This study implemented and evaluated the Hypertension Management Program (HMP), which was originally developed in a high-resource health setting, in a health system with fewer resources and a patient population disproportionately affected by hypertension. Our objectives were to describe how a health system could adapt HMP to meet their needs and calculate total program costs. HMP uses a team-based, patient-centered approach involving clinical pharmacists who contribute to managing patients who have hypertension and ultimately preventing premature death due to uncontrolled hypertension. HMP has 10 components (e.g., EHR patient registries and outreach lists, no copayment walk-in blood pressure checks). Our project involved implementing the key components of HMP in a federally qualified health center (FQHC) in South Carolina. Adaptations from the key components of HMP were made to fit the participants’ settings. A mixed-methods evaluation assessed implementation processes, program costs, and implementation facilitators and barriers. From September 2018 to December 2019, clinical pharmacists conducted 758 hypertension management visits (HMVs) with 316 patients with hypertension. Total program costs for HMP were $325,532 overall and $16,277 per month. Monthly cost per patient was $3.62. The high engagement among clinical pharmacists, along with provider engagements, followed up by the subsequent referral of patients to HMP, facilitated the implementation process. Staff members observed improvements in hypertension control, which increased participation buy-in. Barriers included staff turnover, the perception among some providers that HMP took too much time, as well as perception of HMP as a pharmacy-specific initiative. A team-based, patient-centered approach to hypertension management can be adapted for FQHCs or similar settings that serve patient populations disproportionately affected by hypertension.

**Supplementary Information:**

The online version contains supplementary material available at 10.1007/s11121-023-01529-x.

## Introduction

An estimated 116 million American adults (47.3%) have hypertension, and 92.1 million do not have their hypertension under control (Centers for Disease Control and Prevention [CDC], 2021a). Hypertension contributes to more than 1100 deaths per day and costs the nation an estimated adjusted annual incremental cost of $131 billion per year higher for the hypertensive adult population compared with the non-hypertensive population (Kirkland et al., 2018). Hypertension disproportionately affects people with low income, covered by public insurance, and with no insurance (Leng et al., 2015).Compared to non-Hispanic White and Hispanic persons, African American persons are more likely to develop high blood pressure, develop it at an earlier age, and experience worse outcomes (Thomas et al., 2018). Racial and ethnic disparities in hypertension are persistent, influenced by many factors, and may be better addressed by multicomponent efforts to modify social determinants of health alongside health system interventions. Addressing social determinants of health and modifying health system interventions may prevent premature death related to uncontrolled hypertension (Havranek et al., 2015).

Kaiser Permanente Colorado’s (KPCO) Hypertension Management Program (HMP) is a team-based, patient-centered, integrated care model that aims to improve the diagnosis, treatment, and control of hypertension. In 2009, the Centers for Disease Control and Prevention’s (CDC) Division for Heart Disease and Stroke Prevention (DHDSP) evaluated KPCO’s HMP and found that the program improved health system-wide blood pressure control from 61% in 2008 to 78% in 2010 and 83% in 2012 (CDC, n.d.). Another implementation study demonstrated that selected components of KPCO’s hypertension management intervention could be adapted to safety net settings and lead to improvements in hypertension control among disparate populations (Fontil et al., 2018).

Team-based care approaches are effective at improving hypertension control, are considered cost-effective, and have been used in clinical practice to reduce racial disparities in hypertension outcomes (Bartolome, 2016; Jacob et al., 2015; Proia et al., 2014). Evidence suggests that adding a clinical pharmacist to the primary care team to assist with medication therapy management can address barriers to hypertension treatment and control, particularly in federally qualified health center (FQHC) settings (Rodis et al., 2019). DHDSP replicated and evaluated KPCO HMP in an FQHC that serves a population disproportionally affected by health and healthcare disparities to identify intervention adaptations and costs and determined hypertension control could be improved, the results of which indicated that across FHC in all months, 53.4% of patients had controlled hypertension during the pre-intervention period and 57.3% had controlled hypertension after the intervention was implemented (*p* < 0.01). Statistically significant increases in hypertension control rates were observed in 6 of the 7 clinics (*p* < 0.05). Using this measure, hypertension control rates also increased in the additional clinic, but this increase was not statistically significant at the 5% level. More information regarding health outcome results is available through other publication (CDC, 2021b).

CDC identified an FQHC for replication because the patients they serve have higher rates of chronic disease and premature death. Since their inception in the mid-1960s FQHCs, also referred to as community health centers, have been at the forefront of providing healthcare to patients who are poor, uninsured and reside in areas where accessing care presents a challenge. Like the patients they serve, FQHCs traditionally lack access to adequate funding but work to close the mortality gap and prevent premature death through the maintenance and control of chronic disease (Smith et al., 2017). FQHCs employ the patient-centered medical home (PCMH) model which provided a promising jumping off point for implementing the HMP (HRSA, 2022).

## Purpose and Objectives

CDC contracted with NORC at the University of Chicago (NORC) to adapt, implement, and evaluate the HMP within an FQHC. CDC and NORC selected a health system to partner in program replication using a systematic screening and assessment process adapted from an established methodology developed by Leviton and Gutman (2010). From March 2018 through December 2019, CDC and NORC used a mixed-methods evaluation including review of program documents, qualitative interviews, and microcosting to evaluate implementation processes, facilitators and barriers, and the values of resources used to implement the HMP in an FQHC. This type of implementation and evaluation had not been previously conducted within an FQHC and specifically one in the rural southeast. Understanding implementation and adaptation at an FQHC can provide steps for implementation at other FQHCs to better treat hypertension. The following evaluation questions were used to evaluate implementation and cost:

- To what extent was HMP implemented at FHC as intended?
- What was FHC’s experience with implementing HMP?
- What were the estimated costs of implementing HMP at FHC?

## Intervention Approach

The HMP uses a team-based, patient-centered approach that engages clinical pharmacists to manage patients with hypertension and includes ten components that were identified through the KPCO evaluation as key elements needed for program effectiveness (Table 1) (CDC, n.d.).

**Table 1:** Hypertension Management Program components

**Component**	**Summary of component**
**1. Integrated care team**	Utilizing an integrated care team to educate patients, identifying risk factors for disease, and prescribing and modifying treatments collaboratively. It also includes identifying a program champion, as well as a hypertension governance council
**2.** **Patient registries and outreach lists in the electronic health record (EHR)**	Creating a patient registry and conducting outreach for the Hypertension Management Program
**3.** **No-copayment walk-in/scheduled blood pressure checks**	Implementing blood pressures checks that do not require a copayment or appointment
**4.** **EHR alerts for blood pressure re-checks**	Implementing EHR alerts when blood pressure readings are high. These alerts prompt staff to perform re-checks
**5.** **Education for nurses and other staff on blood pressure measurement technique**	Identifying a blood pressure measurement technique, and providing education to staff to follow best practices for taking blood pressure measurements
**6.** **Promote use of combination medications to treat high blood pressure**	Developing a policy for prescribing combination medications to improve patient medication adherence
**7.** **Hypertension management visits**	Clinical pharmacists developing medication management plans who are approved by the primary care provider and implemented by nurses at hypertension management visits. During these visits, clinical staff provide patient education to increase medication adherence, promote home blood pressure monitoring, and increase awareness of the importance of hypertension control. Clinical pharmacists and other providers are available to patients for consultation hours
**8.** **Promotion of home blood pressure monitoring**	Educating patients on how to use home blood pressure monitors and providing results back to care providers to help them better manage their hypertension
**9.** **Specialty department blood pressure measurements with referral to primary care when needed**	Specialists (such as obstetricians/gynecologists and behavioral health specialists) conducting blood pressure checks and re-checks, and referring patients who have high blood pressure back to their primary care provider
**10.** **Incentives, rewards, and recognition**	Financial and non-financial rewards for high-performing providers and/or teams are based on the achievement of hypertension and overall health system goals

### Selected Intervention Site and Patient Population

CDC and NORC partnered with family health centers (FHCs) to replicate the HMP. FHC is a multi-site FQHC and a Joint Commission–accredited patient-centered medical home (PCMH) that serves five rural counties in South Carolina. FHC operates its main site in the town of Orangeburg and has six full-time satellite sites located throughout the 2423 square mile service area which is home to 135,996 residents (US Census Bureau, 2012). FHC is the sole provider of comprehensive primary and preventive healthcare services in the service area and served 19,250 patients (children and adults) in 2017 (Health Resources & Services Administration, n.d.). All but one clinic houses a retail pharmacy and employs clinical pharmacists. In addition to clinical pharmacists, FHC has clinician providers (medical doctors and family nurse practitioners), nurses, medical office assistants, and a Medicare care coordinator. FHC served 19,250 patients (children and adults) in 2017 (Health Resources & Services Administration, n.d.).

### Implementation of the HMP at FHC

FHC leadership and key staff prepared for implementation throughout the spring and summer of 2018. CDC and NORC provided FHC technical assistance and a toolkit to guide implementation of the ten program components (Table 1). Prior to the start of implementation, there were some activities FHC conducted that were similar to the HMP components. In summer 2018, FHC expanded these components and launched additional components on a rolling basis. The HMP was fully launched on September 5, 2018, and the implementation observation period concluded on December 31, 2019. Table 2 organizes the HMP components by the extent to which they were implemented prior to implementing the HMP and describes key features of FHC’s implementation and adaptation of each HMP component.

**Table 2:** Description of key features of the implementation of the Hypertension Management Program at FHC

**#**	**Hypertension Management Program component**	**Implementation highlights**
**Components not yet implemented at baseline**		
**2**	Patient registries and outreach lists in the electronic health record (EHR)	Clinical pharmacists conducted outreach to patients with uncontrolled hypertension at their last patient encounter, via phone calls
**3**	No-copayment walk-in/scheduled blood pressure checks	Nursing conducted no-copayment blood pressure checks to those who met specified criteria
**4**	EHR alerts for blood pressure re-checks	Information technology (IT) staff programmed an alert to appear in FHC’s EHR as soon as the nurse entered an elevated blood pressure reading
**5**	Education for nurses and other staff on blood pressure measurement technique	Nursing staff training was conducted at the start of the Hypertension Management Program implementation and included step-by-step instructions for taking, reading, and recording blood pressure, as well as information about factors that affect blood pressure
**6**	Promote use of combination medications to treat high blood pressure	FHC created a hypertension medication prescribing protocol based on the report from the Committee appointed to Eighth Joint National Committee.^a^ This protocol also included procedures for follow-up, labs, referrals, hypertension urgency, and hypertension emergency
**7**	Hypertension management visits (HMVs)	Clinical pharmacists developed and implemented medication management plans during HMVs; they were not allowed to titrate medications without provider approval
**Components partially implemented at baseline**		
**1**	Integrated care team	The Associate Director of Pharmacy, who led the Hypertension Coaching program in place before the Hypertension Management Program, moved seamlessly into the program champion role
**8**	Promotion of home blood pressure monitoring	Although home blood pressure monitoring was encouraged prior to the Hypertension Management Program implementation, a wrist blood pressure monitor was provided to all Hypertension Management Program patients at no charge during their second HMV with the clinical pharmacist
**10**	Incentives, rewards, and recognition	FHC rewarded and recognized staff before the Hypertension Management Program, but included meeting specified key goals tied to program implementation metrics, such as the number of blood pressure checks conducted while implementing the Hypertension Management Program
**Components already fully implemented at baseline**		
**9**	Specialty department blood pressure measurements with referral to primary care when needed	FHC focused on encouraging specialists within FHC to refer patients with uncontrolled blood pressure to primary care

^a^FHC defined controlled hypertension as ≤ 140/90 mmHG for all patients regardless of age

FHC adapted several program components to fit their context. For example, under South Carolina state law, pharmacists are unable to enact medication changes without approval from primary care providers. FHC adapted their hypertension management visit (HMV) clinical workflow to allow for provider review of medication management plans prior to each visit with a clinical pharmacist and approval of medication changes afterward. Other adaptations implemented by FHC are described in Table 2.

## Methods

A mixed-methods evaluation assessed implementation processes, facilitators and barriers to implementation, and program costs. Health outcomes of the intervention are presented elsewhere (CDC, 2021b).

The evaluation protocol was exempted from further review by NORC at the University of Chicago’s Institutional Review Board.

### Review of Program Materials and Performance Data

Throughout the implementation period, we conducted weekly technical assistance calls with FHC staff to provide guidance and understand implementation experiences. FHC shared the training materials that they developed for staff, including protocols, clinical process documents, and screenshots of the EHR templates.

On a monthly basis, FHC shared outreach registry reports showing how many patients had been contacted by the clinical pharmacists conducting outreach. FHC patients were eligible for participation in HMP if they were aged 18 to 85 years old with a diagnosis of hypertension (ICD-10 code: I10), and no evidence of excluding conditions such as ESRD, transplant, or pregnancy. We used this information to guide the collection and interpretation of cost and qualitative interview data. We compared this information against each program component as described in the implementation toolkit to assess the extent to which FHC implemented program components as intended. We developed “crosswalks” to collect details on the implementation processes, timing, and staffing associated with each component. We asked the HMP Clinical Coordinator to verify details and provide updates throughout the implementation period.

### Microcosting

We utilized a microcosting approach to estimate incremental costs associated with labor, facilities, supplies, and other resources required for program implementation and to allocate these costs across FHC’s program activities. We estimated costs from the healthcare perspective and used a 0% discount rate over costs measured at different times of the implementation given the 18-month time horizon of the program implementation. Incremental costs represent additional changes in costs resulting from implementation of HMP at FHC above the costs of standard of care. We estimated quantities of services performed for outreach, blood pressure checks, and HMP management plan activities and patient visits from evidence of event occurrences and time stamp information associated with those events contained in the EHR. FHC’s medication management plan process required a primary care provider (PCP) visit prior to the first HMV. We could not definitively identify visits in the EHR specifically conducted for this purpose. Therefore, by assumption, we included the cost of one PCP visit for each patient initiating HMV, valued using the 2020 Medicare facility reimbursement rate for CPT 99,214 in South Carolina ($76.91) (Centers for Medicare & Medicaid Services, n.d.). Furthermore, we did not include a sensitivity analysis because most of the costs are based on direct measurement. While we do include assumed costs, such as reimbursement rates, it is unlikely that realistic levels of uncertainty in these parameters would have a substantial impact on the cost results. However, we acknowledge that our costs are measured with uncertainty and represent estimates as opposed to exact measurement.

We worked with FHC over the course of implementation to develop and complete cost data collection forms and time diaries to obtain information not available in administrative data. Parameters included overhead and labor costs for non-HMV activities (i.e., staff time and wage estimates by staff types), supplies, and materials. Labor costs collected from time diaries in the cost collection forms included hours for project management, IT and training, loaded labor costs including wages, fringe benefits, and overhead. Project management costs included time spent planning by FHC staff, but not additional work conducted by CDC and NORC adapting the intervention to support implementation at FHC. Training included nurse and pharmacist training on the HMP program and additional training on data entry required to capture program data. Hours for outreach were based on time diary estimates of time per outreach activity, multiplied by volume of outreach per person per month generated from administrative data. Other costs collected included ancillary costs such as supplies and incentive payments.

We calculated the total cost of HMP to FHC, the total cost per eligible patient, and the operating cost per eligible patient. We divided these costs by each phase of implementation: (1) pre-implementation (capturing planning and start-up costs), April 2018–August 2018; (2) initial implementation (capturing ramp-up costs): September 2018–March 2019; and (3) full implementation (capturing ongoing costs), April 2019–December 2019.

### Qualitative Interviews of Staff and Patients

We conducted 17 interviews with FHC staff focusing on early implementation experiences in November/December 2018, and 10 interviews with FHC staff in February 2020, after the end of the implementation period. FHC selected relevant staff across sites and various roles to participate in the interviews. Roles represented include clinical (e.g., physicians, nursing, and pharmacy) staff and those in leadership roles, such as the HMP clinical coordinator, chief executive officer (CEO), chief information officer (CIO), and chief medical officer (CMO).

We conducted interviews with 7 FHC patients in February 2020 to learn about their experiences with HMP and blood pressure management. The HMP clinical coordinator worked with clinical pharmacists to purposively recruit patients who attended visits with clinical pharmacists through HMP. All interviewees provided verbal consent.

Using NVivo (QSR International Americas, Burlington, MA), we conducted a content analysis of detailed transcript style notes from interviews with FHC staff and patients. We created an initial list of categories based on evaluation questions and the Consolidated Framework for Implementation Research (Damschrode et al., 2009) domains and drafted a codebook to guide coding of data from interviews. We operationalized the research question and model-based analytic dimensions in the codebook, which provided clear and concise guidelines for categorizing all qualitative data collected. A team of four researchers coded a first set of transcripts together and met to discuss areas where the code application was unclear or inconsistent. After this initial coding, two junior researchers coded the remaining transcripts and met regularly with senior researchers to routinely review codes and discuss themes. This process served to improve the team’s inter-coder reliability and identify any necessary revisions to the codebook. As coding progressed, we refined the categories as key themes emerged. We organized our final analysis into overarching categories (i.e., implementation facilitators and barriers).

## Results

### Implementation Metrics

We analyzed the following process metrics to assess the extent to which HMP was implemented as intended: (1) registry-based patient outreach attempts and scheduled visits; (2) new hypertension management visits; (3) medication management plans and total hypertension management visits; and (4) walk-in/scheduled blood pressure checks. During the implementation period, 4799 HMP-eligible patients had at least one visit with valid blood pressure readings.

**Fig. 1:**
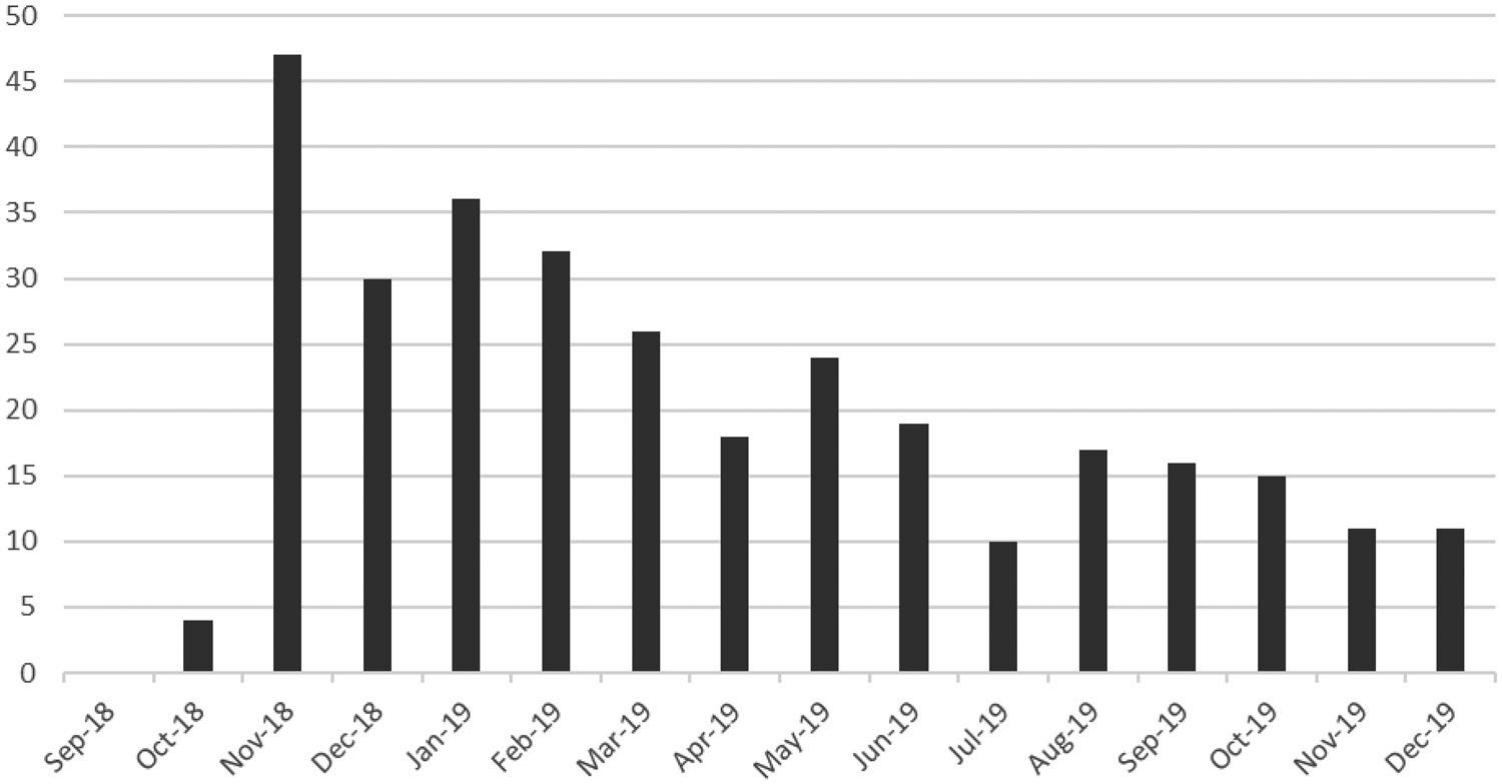
New Hypertension Management Program patients by month

FHC clinical pharmacists made an average of 688 patient contact (phone call) attempts per month, yielding an average of 65 scheduled hypertension management visits per month (21 new and unique patients per month), and 316 new HMP patients attended an HMV with a clinical pharmacist during the intervention period, September 2018 to December 2019 (Fig. 1). The number of new HMP patients was highest in November 2018 (47 patients) and declined throughout 2019.

**Fig. 2:**
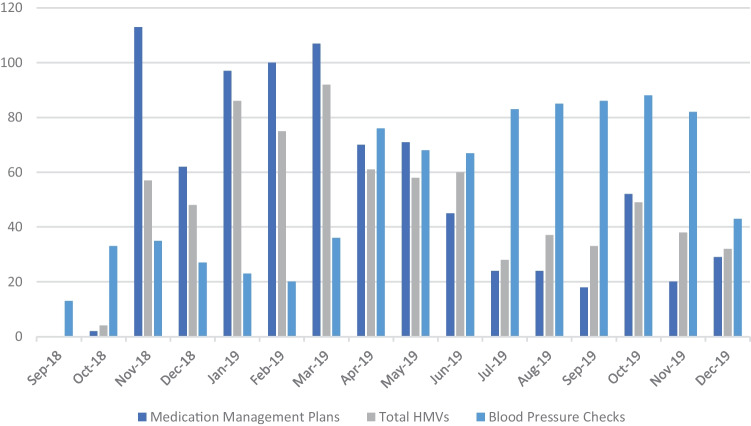
Number of medication management plans developed, total HMVs, and blood pressure checks

Clinical pharmacists obtained provider approval on medication management plans prior to implementing them during hypertension management visits. Clinical pharmacists developed a total of 834 medication management plans and conducted 758 HMV (316 unique patients) (Fig. 2) overall. The number of visits declined in the final quarter of the implementation period. We analyzed the number of no-copayment walk-in/scheduled blood pressure (BP) checks (Fig. 2) as a marker of how many patients were directly engaged with HMP. Overall, FHC staff conducted 865 BP checks (666 unique patients) during the intervention period.

### Cost Results

Total program costs for HMP were $325,532 overall and $16,277 per month across all implementation months (data not shown). The average monthly cost of HMP decreased from $20,788 in the initial implementation period to $14,727 in the full implementation period (data not shown). From June through July 2019, FHC suspended registry and outreach activities due to system-wide IT issues, while other activities continued. Excluding these two months from analysis, monthly costs were $16,561 in the full implementation phase (data not shown).

The monthly cost per patient among 4799 patients eligible for the HMP program was $3.62.The marginal cost of adding additional patients was $3.07 per patient per month. Table 3 shows the per patient cost of HMP implementation at FHC.

**Table 3:** Per-person cost of the Hypertension Management Program

	**Total cost per patient**	**Monthly cost per patient**	**Marginal monthly cost per new patient**
All eligible patients (*n* = 4799)	$67.83	$3.62	$3.07
Patients with HMV (*n* = 316)	$1,030.17	NA	NA

HMV = hypertension management visit

**Fig. 3:**
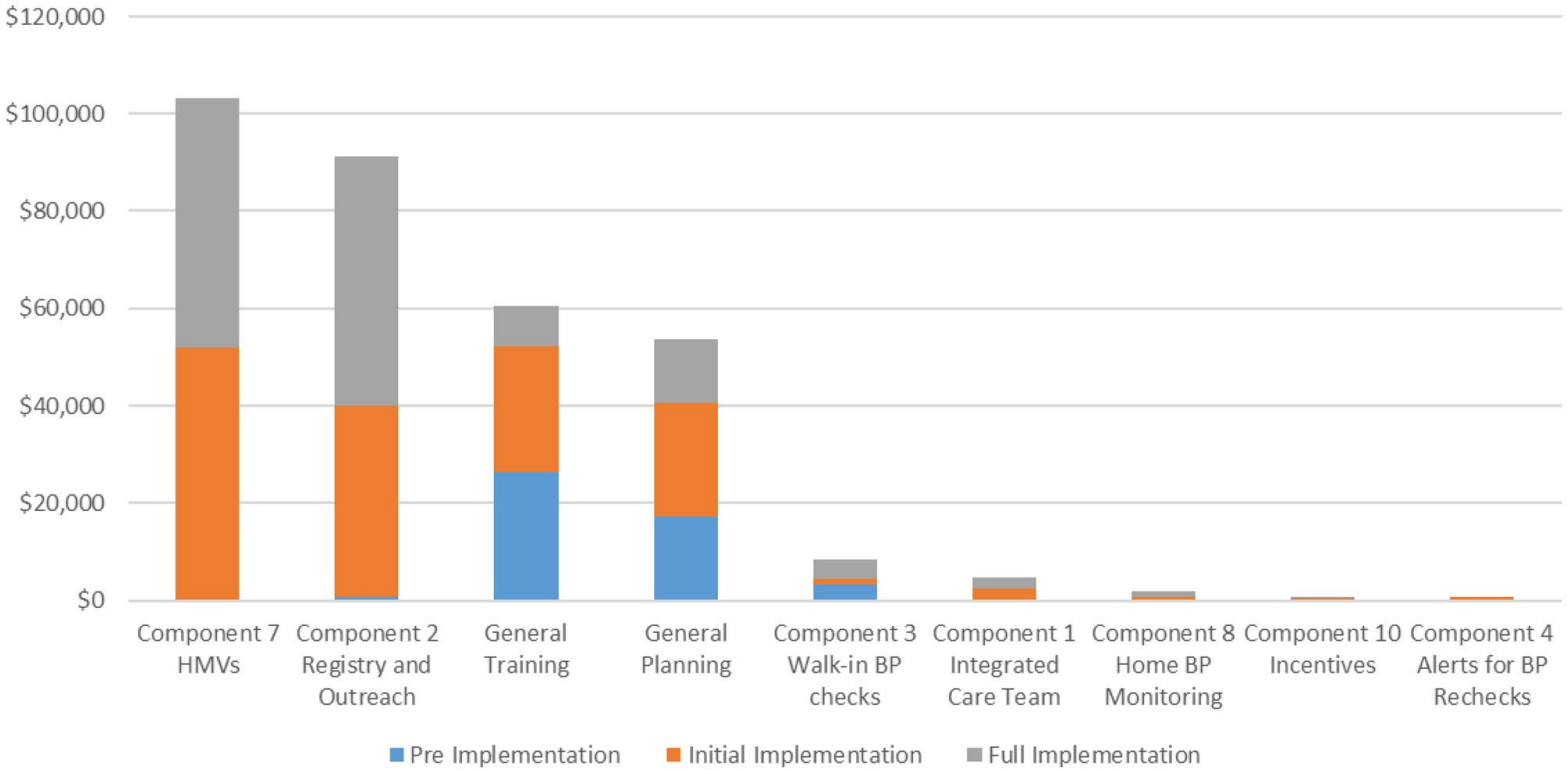
Costs by Hypertension Management Program component and activity

HMP costs by activity and phase are listed in Fig. 3 and eTable 1. HMVs (HMP Component 7) were the highest cost component at $103,285. Of this total, $44,743 (43%) were directly related to cost for the 758 HMVs conducted at FHC. This included $37,960 in labor costs for pharmacists conducting the visit and $6783 in labor costs for nurses doing intake and workups. Additional costs for the HMVs were for developing and reviewing medication management plans cost an additional $33,495 (32% of total HMV visit costs).

FHC conducted 19 HMP Q&A visits (i.e., introductory HMVs), which were short visits with a pharmacists that did not require physician provider time or development of a medication management plan, and incurred $743 in costs. FHC’s HMP clinical workflow required patients to have a primary care physician (PCP) visit before referral to HMVs, which cost a total of $24,304, although it is likely that most or all of these costs would be reimbursable.

Patient registries and outreach (HMP Component 2) comprised the second highest cost of any component or activity area ($90,688). Of this, $74,812 was due to labor hours for outreach. Outreach was conducted by a single staff-member in the first month of implementation. Beginning in October 2018, outreach was conducted by nine pharmacists, typically one per satellite clinic and two for the main facility. Registry-based outreach incurred $9402 in IT labor associated with creating and maintaining the patient registries, and $6474 in labor costs for the project manager to supervise outreach.

The next highest cost areas were training ($60,544) and general planning activities ($53,722). These activities spanned all facets of program implementation and were not allocated to specific components. FHC conducted 10 staff training events for HMP and reported the duration and attendance of each training session by job type. Planning activities included non-allocated labor time for the project manager and IT staff, as well as meeting costs for FHC’s hypertension management team and the HMP council.

FHC conducted at least 865 walk-in blood pressure checks (Component 3) at a total estimated cost of $5143 and spent $3200 on advertising for a total cost of $8343. Integrated care team activities (Component 1) cost an estimated $4832. This cost includes 10 pharmacist care team meetings over the course of the implementation period. HMV medication management plan team activities are included in HMP Component 7. No other activity area comprised more than 1% of the total cost.

### FHC Staff and Patient Experiences with HMP

Overall, FHC staff and patients expressed positive views about their experiences with HMP. They identified several HMP implementation facilitators and barriers (Table 4.)

**Table 4:** Hypertension Management Program Implementation Facilitators and Barriers, FHC, South Carolina

**Implementation facilitators**	**Implementation barriers**
**▪ High engagement among clinical pharmacists from the start of implementation**	▪ Perception among some providers that the Hypertension Management Program took too much time, which translated to less Hypertension Management Program referrals at some sites
**▪ Provider engagement and the subsequent referral of patients to the Hypertension Management Program**	▪ Staff turnover, particularly among FHC leadership, during the implementation
**▪ Staff members’ observation of improvements in patient hypertension control and the corresponding increased buy-in**	▪ View among some staff that Hypertension Management Program was a siloed, pharmacy-specific initiative
**▪ Stable leadership from the Hypertension Management Program clinical coordinator throughout the implementation**	▪ Time needed to conduct registry-based outreach

#### Implementation Facilitators

##### Clinical Pharmacists Were Highly Engaged in HMP from the Start of Implementation 

In both rounds of interviews, pharmacists expressed enthusiasm for their augmented role in managing patients’ hypertension medications. As the HMP clinical coordinator stated, “Although we were already doing hypertension coaching, [HMP] pushed the door open even further for clinical pharmacists here at FHC. I think it went pretty well, and it gave providers (clinicians) who had not worked with clinical pharmacists before the opportunity to do so.” In turn, FHC patients reported being receptive to hypertension management visits. One patient said, “I wondered why I was going to a pharmacist because I never talked to one before, except when I sometimes had question about a medication. It was a good experience, though.”

##### FHC Staff Described Provider Engagement as an Important Facilitator of Successful Implementation

In particular, pharmacy staff described how provider engagement—and the subsequent referral of patients to HMP—was key: “For the sites that were truly successful, those pharmacists who saw a lot of referrals and saw a lot of HMP patients [*sic*], it was due to that provider buy-in.” Providers who were engaged in HMP viewed pharmacy staff as resources for improving patient care and reducing their workloads. In the baseline implementation interviews, providers reported being receptive to working with clinical pharmacists, noting appreciation for their expertise. During the final implementation interviews, these providers described how they established open and frequent communication with pharmacists and found a workflow that helped them better manage patients’ medications in collaboration with pharmacy staff.

HMP has worked tremendously great at bringing patients’ blood pressure down to goal. The collaboration with me and the pharmacist… it helps patients feel relieved that they have someone who really cares about them who is working to bring their blood pressure down [*sic*]. The pharmacist goes over medications and helps with diet. We try to bring patients back every week until we can get them at goal. We have seen tremendous control in our patients with their blood pressure who were participants [in HMP].—Provider

Providers viewed HMVs as beneficial because they could spend less time focusing on hypertension management during short (e.g., 15 min) primary care visits when they needed to address other health related issues.

##### FHC Staff Saw Improvements in Patient Hypertension Control, Leading to Further Staff Buy-In

Through targeted medication management and education on lifestyle factors, clinical pharmacists were able to help patients achieve blood pressure control. As one patient stated about the pharmacist, “She tells me my meds and what I can expect… the blood pressure pill has the water pill in it now. [My medication] is working out great. She helped me figure out how many times a day and when to take it in the morning.” Several patients who achieved blood pressure control through HMP felt that clinical pharmacists were accessible and appreciated them for their ability of being easy to reach out to them with questions. Patients described the critical role of patient education, home blood pressure monitoring, and medication adherence promoted by clinical pharmacists through HMP.

Because clinical pharmacists were able to spend more time with patients during hypertension management visits relative to regular primary care visits, they were able to develop deeper relationships with patients. As one pharmacist stated, “[Patients] are used to seeing me fill their prescriptions, but they like having the opportunity to spend more time talking to me about their medications. [It gives] me more time to explain to them why certain changes were made to their medications. I think it empowers them to watch their blood pressure even more and be more cognizant of what affects blood pressure. I think that [HMP] helped with altering their lifestyle in a way where they try to do a little more to bring their blood pressure down.”

##### The HMP Clinical Coordinator Provided Stable Leadership for HMP Implementation Throughout the Course of the Implementation Period

In the final implementation interviews, pharmacy staff reported that regular meetings with the HMP clinical coordinator to discuss metrics on HMVs and outreach were particularly helpful in advancing HMP. As one member of the administrative staff reported during the final interviews, the HMP clinical coordinator effectively leveraged HMP leadership meetings for identifying challenges, assigning tasks to HMP leadership staff, and following up with these staff to ensure accountability throughout the implementation.

#### Implementation Barriers

##### The Perception That HMP Took Too Much Time Hindered Buy-In from Some Providers, Which Translated to Lower HMP Referrals at Some Sites

During early implementation interviews, providers and pharmacists reported that workflows associated with HMP took additional time. Pharmacists indicated that the lack of an established collaborative practice agreement sometimes created a cumbersome process that slowed provider buy-in. While some pharmacists and providers were able to find workflows that helped them better manage patients, others remained less engaged. One pharmacist noted that a provider who viewed HMP as extra work referred patients to HMVs at a lower rate compared to another provider who did not view HMP as a time burden.

## Turnover Among FHC Staff, Particularly Among FHC Leadership, Was a Primary Implementation Barrier

During early implementation interviews, we learned that transitions among nursing leadership had led to lower levels of buy-in among nursing staff. Throughout the rest of the implementation period, turnover among other leadership across the organization occurred as well; there were two separate CMO transitions, one transition among CIOs, and one transition among pharmacy department leadership. The HMP clinical coordinator noted that turnover among the CMO was particularly problematic for HMP implementation, given that this administrative staff member provided leadership for all clinical staff, including the pharmacy department. Turnover among clinical staff was also reported as a barrier to HMP implementation. It should be noted that turnover was also a barrier to health outcome data collection as the staff responsible for data abstraction changed at a critical time of study data collection and analysis.

## Some Staff Viewed HMP as a Siloed, Pharmacy-Specific Initiative

The HMP clinical coordinator described how some staff erroneously viewed HMP as strictly a pharmacy program. Providers and administrative staff echoed this perception and attributed lower levels of buy-in among providers and nursing staff to this view. The HMP clinical coordinator said it would have been ideal to not only have leadership from each department committed and engaged from the beginning, but also one or more clinical staff members from other departments who could co-champion the program along with a pharmacy champion. While having leadership involved would have been important, having “someone on the ground” as a champion would have been equally valuable.

## Pharmacy Staff Reported That the Time Needed to Conduct Outreach Was a Major Implementation Barrier

In early implementation interviews, pharmacy staff reported challenges engaging patients through outreach. One of the largest challenges was incorrect patient contact information. Pharmacy staff said that conducting outreach was very time consuming and difficult to fit in with other responsibilities. In the final implementation interviews, two pharmacists said that referring patients at the point of care would have been preferable to conducting outreach to them after they had recently been seen.

## Discussion and Conclusion

The results of this HMP replication and evaluation may not be generalizable to all health systems, but offer considerations for public health practice and preventative care. The setting and patient populations for the replication were substantially different from the original implementation of HMP at KPCO. Hypertension is more prevalent in the southeastern region of the USA; in South Carolina, for example, 38.1% of adults reported that they have a diagnosis of hypertension, whereas self-reported prevalence rates in the northwestern portion of the USA range from 24.3 to 30%, and 25.9% in Colorado specifically (CDC et al., 2015). According to HRSA (2021), the median household income is $34,943 in Orangeburg County, South Carolina, whereas in the KPCO sample, the median household income was approximately $66,500 per year. KPCO served a population that was predominantly White, medically insured, and with only small proportions of minority populations. In contrast, FHC served a population in which 89% were Black/African American and 21% were uninsured and in 2017, 86% had incomes that were at or below the 100% Federal Poverty Guideline. Despite these differences and short timeline for implementation (16 months), FHC was able to see positive changes in hypertension care delivery by implementing KPCOs HMP, adapting components to fit their unique health system’s needs.

The quantitative and qualitative results suggest that although there are barriers to implementing significant quality improvement changes when working at healthcare facilities that serve the under- and uninsured, at FHC there was a desire among staff to stretch and adapt proven initiatives to better serve their patients. The two most costly components of the implementation were the HMVs and the registry outreach. Previously, these activities had been outside the scope of work of FHC pharmacists. The labor costs associated with pharmacists doing registry outreach was high; however, the data does not show that pharmacists were working overtime to complete this activity, so these costs were not over normal pharmacy costs to FHC. Furthermore, the results suggest that HMVs were an integral part of patients’ success, providing evidence that pharmacists’ involvement in patient care had a direct impact on blood pressure control.

The success of the implementation utilizing pharmacists suggests that collaborative practice agreements could enable clinical pharmacists who can assist in providing clinical care to patients to relieve provider burden when working in a setting where providers may be stretched regarding patient load. Even when a robust team exists to implement team-based care initiatives, the team and patients can benefit by having collaborative practice agreements that allow pharmacists to work at the top of their license to adjust patient medication without having to confer with providers. Having organizational policies in place that allow staff to work at the top of their licensure and provide the flexibility to establish clinical workflows that engage all available medical professionals works to the advantage of patients and relieves burden on the team.

Some limitations to the FHC HMP implementation that should be considered when deciding whether to adapt include the number of pharmacists employed by FHC which may be much larger than at other FQHCs so identifying staff to conduct hypertension management visits could be more challenging and present the need for a different implementation plan. Also, to be conservative regarding the potential costs of HMP, we assigned the 2020 Medicare fee-for-service (FFS), S.C. reimbursement rate for procedure code 99,214 cost to pre-HMV primary care visits because we were not able to uniquely identify these visits in the EHR and because facility level reimbursement rates were not available. However, many of these visits may not have occurred, or when they did occur the reimbursement rate may have been lower or different from the Medicare FFS rate. FQHCs typically receive payment from varied sources including Medicaid and Medicare Prospective Payment Systems so it is difficult to precisely know the reimbursement rate for primary care visits precisely. It would benefit new implementations to include a cost-effective analysis to understand the true cost of implementation based on adaptations made during implementation.

Strategies for program implementation and adaptation emerged throughout the implementation of HMP at FHC. Utilizing clinical pharmacists currently serving in a retail capacity in a more clinical role allows the healthcare system to improve their ability to serve patients. Empowering hypertension champions across clinical teams has a direct impact on the ability of the team to affect change in the hypertension management of patients. Adapting the HMP to fit a given setting by deciding who would be best to serve in needed roles that could include hypertension management visits or registry outreach is integral to successful implementation. Opportunities for further exploration could include a focus on recruitment and referral of patients which remained low throughout FHC implementation, as well as adapting HMP for telehealth which could increase hypertension management visits and overall contact with patients in future attempts at replication or adaptation. Those interested in adaptation should consider their staff resources, readiness for organizational change, and considerations regarding staff time and cost as they consider implementation. Evaluation of new implementation should include the collection of systematic data related to adaptation in hopes to identify adaptations that can be generalized across FQHCs. Putting the core components of HMP into practice at an FQHC requires adaptation and flexibility and may be a viable approach in other care settings where pharmacists or other medical professionals who can titrate medication are included as part of the healthcare team.

### Supplementary Information

Below is the link to the electronic supplementary material.

Supplementary file1 (DOCX 18 KB)
