# Correction: A Mixed-Methods Approach for Evaluating Implementation Processes and Program Costs for a Hypertension Management Program Implemented in a Federally Qualified Health Center

**DOI:** 10.1007/s11121-024-01670-1

**Published:** 2024-03-20

**Authors:** Aisha Tucker-Brown, Michelle Spafford, John Wittenborn, David Rein, Ashley Marshall, Kincaid Lowe Beasley, Marla Vaughan, Natalie Nelson, Michelle Dougherty, Roy Ahn

**Affiliations:** 1 Division for Heart Disease and Stroke Prevention, Centers for Disease Control and Prevention, 4770 Buford Highway NE, GA 30341 Atlanta, USA; 2 Health Care Evaluation Department, NORC at the University of Chicago, 4350 East-West Highway, 8th Floor, MD 20814 Bethesda, USA; 3 Public Health Department, NORC at the University of Chicago, 55 East Monroe, 31st Floor, IL 60603 Chicago, USA; 4 Public Health Department, NORC at the University of Chicago, 1447 Peachtree Street NE, Suite 700, Atlanta, GA 30309 USA; 5 Division of Laboratory Systems (DLS), Centers for Disease Control and Prevention, 2400 Century Center, Atlanta, GA 30345 USA; 6 Family Health Centers, Inc, 3310 Magnolia St, Orangeburg, SC 29115 USA; 7 Public Health Department, NORC at the University of Chicago, 4350 East-West Highway, 8th Floor, MD 20814 Bethesda, USA

**Correction to: Prevention Science** 10.1007/s11121-023-01529-x

The article “A Mixed-Methods Approach for Evaluating Implementation Processes and Program Costs for a Hypertension Management Program Implemented in a Federally Qualified Health Center”, written by Tucker-Brown, A., Spafford, M., Wittenborn, J., Rein, D., Marshall, A., Beasley, K.L., Vaughan, M., Nelson, N., Dougherty, M., and Ahn, R., was originally published electronically on the publisher’s internet portal on 30 June 2023 without open access. With the author(s)’ decision to opt for Open Choice the copyright of the article changed on 16 February 2024 to © The Author(s) 2023 and the article is forthwith distributed under a Creative Commons Attribution 4.0 International License, which permits use, sharing, adaptation, distribution and reproduction in any medium or format, as long as you give appropriate credit to the original author(s) and the source, provide a link to the Creative Commons licence, and indicate if changes were made. The images or other third party material in this article are included in the article’s Creative Commons licence, unless indicated otherwise in a credit line to the material. If material is not included in the article’s Creative Commons licence and your intended use is not permitted by statutory regulation or exceeds the permitted use, you will need to obtain permission directly from the copyright holder. To view a copy of this licence, visit http://creativecommons.org/licenses/by/4.0/.

The original article has been corrected.

