# Supplementary material for: A Mixed-Methods Approach for Evaluating Implementation Processes and Program Costs for a Hypertension Management Program Implemented in a Federally Qualified Health Center
Source: Prev Sci. 2023 Jun 30;25(Suppl 1):10–21. doi: 10.1007/s11121-023-01529-x (PMC11133121; doi:10.1007/s11121-023-01529-x)
Supplement: Supplementary file 1 — Supplementary file1 (DOCX 18 KB) [file 11121_2023_1529_MOESM1_ESM.docx]

**Supplementary information**

**eTable 1.** Detailed Cost Information

|  | **Pre implementation** | **Initial implementation** | **Full implementation** | **Total** |
| --- | --- | --- | --- | --- |
| **HMP visits** | **$0** | **$51,918** | **$51,366** | **$103,285** |
| **Registry and outreach** | **$695** | **$39,283** | **$51,405** | **$91,383** |
| **General training** | **$26,425** | **$25,799** | **$8,320** | **$60,544** |
| **General planning** | **$17,150** | **$23,407** | **$13,165** | **$53,722** |
| **Walk-in BP checks** | **$3,200** | **$1,123** | **$4,020** | **$8,343** |
| **Integrated care team** | **$0** | **$2,347** | **$2,485** | **$4,832** |
| **Home BP monitoring** | **$0** | **$651** | **$1,331** | **$1,982** |
| **Incentives** | **$0** | **$350** | **$450** | **$800** |
| **Alerts for BP rechecks** | **$0** | **$642** | **$0** | **$642** |
| **Total** | **$47,470** | **$145,519** | **$132,543** | **$325,532** |
| **Per-month** |  | **$20,788** | **$14,727** | **$16,277** |
